# Human Intravenous Immunoglobulin Alleviates Neuropathic Symptoms in a Rat Model of Paclitaxel-Induced Peripheral Neurotoxicity

**DOI:** 10.3390/ijms22031058

**Published:** 2021-01-21

**Authors:** Cristina Meregalli, Laura Monza, Alessia Chiorazzi, Carla Scali, Chiara Guarnieri, Giulia Fumagalli, Paola Alberti, Eleonora Pozzi, Annalisa Canta, Elisa Ballarini, Virginia Rodriguez-Menendez, Norberto Oggioni, Guido Cavaletti, Paola Marmiroli

**Affiliations:** 1 Experimental Neurology Unit, School of Medicine and Surgery, and NeuroMI (Milan Center for Neuroscience), University of Milano-Bicocca, 20900 Monza, Italy; cristina.meregalli@unimib.it (C.M.); laura.monza@unimib.it (L.M.); alessia.chiorazzi@unimib.it (A.C.); giulia.fumagalli1@unimib.it (G.F.); paola.alberti@unimib.it (P.A.); eleonora.pozzi@unimib.it (E.P.); annalisa.canta@unimib.it (A.C.); elisa.ballarini@unimib.it (E.B.); virginia.rodriguez1@unimib.it (V.R.-M.); norberto.oggioni@unimib.it (N.O.); paola.marmiroli@unimib.it (P.M.); 2 Global Medical and R&D Department, Kedrion S.p.A., Località Ai Conti, Castelvecchio Pascoli, 55051 Lucca, Italy; C.Scali@kedrion.com (C.S.); C.Guarnieri@kedrion.com (C.G.); 3 Department of Biotechnology and Biosciences, University of Milano-Bicocca, 20126 Milan, Italy

**Keywords:** paclitaxel, neuropathic pain, intravenous immunoglobulin (IVIg), chemotherapy, axon degeneration, IENF

## Abstract

The onset of chemotherapy-induced peripheral neurotoxicity (CIPN) is a leading cause of the dose reduction or discontinuation of cancer treatment due to sensory symptoms. Paclitaxel (PTX) can cause painful peripheral neuropathy, with a negative impact on cancer survivors’ quality of life. While recent studies have shown that neuroinflammation is involved in PTX-induced peripheral neurotoxicity (PIPN), the pathophysiology of this disabling side effect remains largely unclear and no effective therapies are available. Therefore, here we investigated the effects of human intravenous immunoglobulin (IVIg) on a PIPN rat model. PTX-treated rats showed mechanical allodynia and neurophysiological alterations consistent with a severe sensory axonal polyneuropathy. In addition, morphological evaluation showed a reduction of intra-epidermal nerve fiber (IENF) density and evidenced axonopathy with macrophage infiltration, which was more prominent in the distal segment of caudal nerves. Three weeks after the last PTX injection, mechanical allodynia was still present in PTX-treated rats, while the full recovery in the group of animals co-treated with IVIg was observed. At the pathological level, this behavioral result was paralleled by prevention of the reduction in IENF density induced by PTX in IVIg co-treated rats. These results suggest that the immunomodulating effect of IVIg co-treatment can alleviate PIPN neurotoxic manifestations, probably through a partial reduction of neuroinflammation.

## 1. Introduction

Paclitaxel (PTX) is a very effective anti-tubulin drug belonging to the family of taxanes. It is largely employed in the treatment of many solid tumors including breast, prostate, non-small cell lung, pancreatic and gynecological cancers [1]. Despite its efficacy, its use is often limited by the onset of PTX-induced peripheral neurotoxicity (PIPN), a common and potentially severe side-effect occurring in up to 87% of patients undergoing PTX chemotherapy regimen [2]. PIPN is characterized by a distal-to-proximal nerve degeneration pattern and by the so called “PTX associated acute pain syndrome (PAPS)”, which has been suggested to be closely linked to the development of chronic PIPN [3] and is considered to be a specific type of neuropathic pain typical of PIPN [4]. There is no available prevention strategy for PIPN, and its treatment is also problematic. To better understand the mechanisms underlying PIPN, several animal models have been developed over the years [5,6,7], reaching a high level of reproducibility and similarity with the clinical pictures observed in patients undergoing PTX-based chemotherapy.

Despite remarkable efforts at the preclinical level, a comprehensive knowledge of the mechanisms leading to PIPN is still lacking. PTX dependent inhibition of tubulin depolymerization leading to microtubule dysfunction seems to be the most reasonable hypothesis [8], although dysfunction of calcium channels [9] as well as the activation of toll like receptor 4 (TLR4) [10] could also be involved. However, in the last decades, the investigation of the role of neuroinflammation in the onset of chemotherapy-induced peripheral neurotoxicity (CIPN) has gained increasing interest, especially in PIPN [11].

In fact, several studies reported an increase of pro-inflammatory cytokines and chemokines in the plasma, serum, dorsal root ganglia (DRG) neurons, sciatic nerves, skin of the hind paw and spinal cord of PTX-treated rodents [12]. These alterations in the chemokines and cytokines profiles were associated with macrophage infiltration in DRG and sciatic nerves [13,14,15,16,17] and glial activation in the central and peripheral nervous system [18,19,20]. In particular, it has been observed that PTX induces the upregulation of TLR4 that, in turns, leads to the recruitment and activation of macrophages with a M1 phenotype in DRGs, triggering the release of pro-inflammatory mediators [14,21,22], while macrophage infiltration in sciatic nerves seem to follow axonal damage [23]. In this context, activated glial cells may contribute to the release of cytokines and chemokines exacerbating the inflammatory response. Moreover, PTX treatment induces the activation of Nod-like receptor 3 inflammasome (NLRP3), which is an essential component of the inflammatory response [24]. In the last years, the effectiveness of immunomodulatory drugs in the prevention of pain-like behavior in rodent models of PIPN has been reported [20,25,26,27,28,29,30,31]. In fact, the inhibition of the pro-inflammatory cascade initiated by IL-20 through the administration of an anti-IL-20 monoclonal antibody prior to PTX treatment, attenuated not only the nocifensive behavior, but also peripheral nerve damage in experimental PIPN [14]. These data suggest that immunotherapeutic strategies targeting the inflammatory response may be effective in the management of CIPN.

Human intravenous immunoglobulin (IVIg) are therapeutic polyspecific IgGs derived from plasma pools of thousands of healthy donors, characterized by multiple immunomodulatory and anti-inflammatory properties. IVIg are used to manage neuropathic pain from various neurological disorders [32]. While their effectiveness has been demonstrated in several animal models of autoimmune and inflammatory neuropathies [33,34] mimicking Guillain-Barrè syndrome or chronic inflammatory demyelinating polyneuropathy [35] and also in experimental bortezomib-induced peripheral neuropathy [36], their use in PIPN has not yet been investigated. Therefore, the aim of this study was to evaluate the effects of human IVIg in an established and well-characterized rat model of peripheral neuropathy and neuropathic pain induced by PTX.

## 2. Results

### 2.1. Safety and Tolerability of PTX and PTX + IVIg Co-Treatment

PTX and PTX-IVIg co-treatment were well tolerated by the animals during the experiment, it did not induce any significant difference in body weight compared to vehicle-treated rats and no animals died during the study nor showed signs of distress.

**Figure 1:**
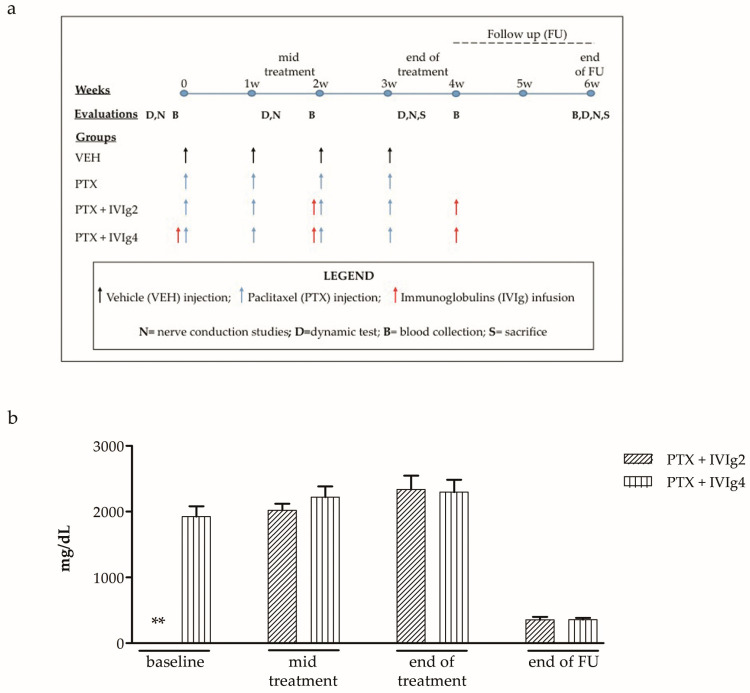
Flow chart and serum concentration of human intravenous immunoglobulin (IVIg). (**a**) Flow chart of the experimental plan. (**b**) At baseline, mid treatment and at the end of treatment blood samples were collected 1 h after IVIg infusion. At follow up, blood samples were collected two weeks after the last IVIg infusion. ** infusion with IVIg not performed as per the protocol.

Serum concentrations of IVIg were measured in each IVIg-treated group over the entire sampling period, i.e., until follow up in all animals (Figure 1a). At each time point, no significant difference in the concentration of IVIg between the two groups was observed (Figure 1b).

### 2.2. IVIg Significantly Attenuates Mechanical Allodynia

**Figure 2:**
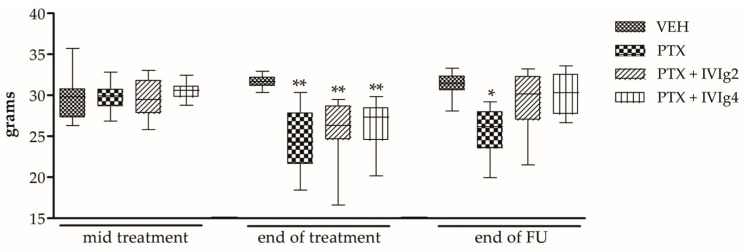
Effects of PTX and IVIg co-treatment on mechanical allodynia. IVIg alleviates mechanical allodynia in a rat model of PIPN at the end of FU. * *p* < 0.01, ** *p* < 0.001 vs. VEH (*n* = 12 rats/group). The statistical analysis was performed using the Kruskal-Wallis test.

At baseline, no significant difference in the mean withdrawal threshold was observed among the groups. At the end of the treatment, PTX, PTX + IVIg2, PTX + IVIg4 groups showed a significant decrease in the mechanical threshold compared to VEH-treated rats (*p* < 0.001; Figure 2), indicating the development of mechanical allodynia. After the follow up period, only the group treated with PTX alone showed persisting mechanical allodynia (*p* < 0.01; Figure 2). The values observed in both IVIg co-treated groups were not different from the values obtained by testing VEH-treated rats.

### 2.3. Nerve Conduction Studies

**Figure 3:**
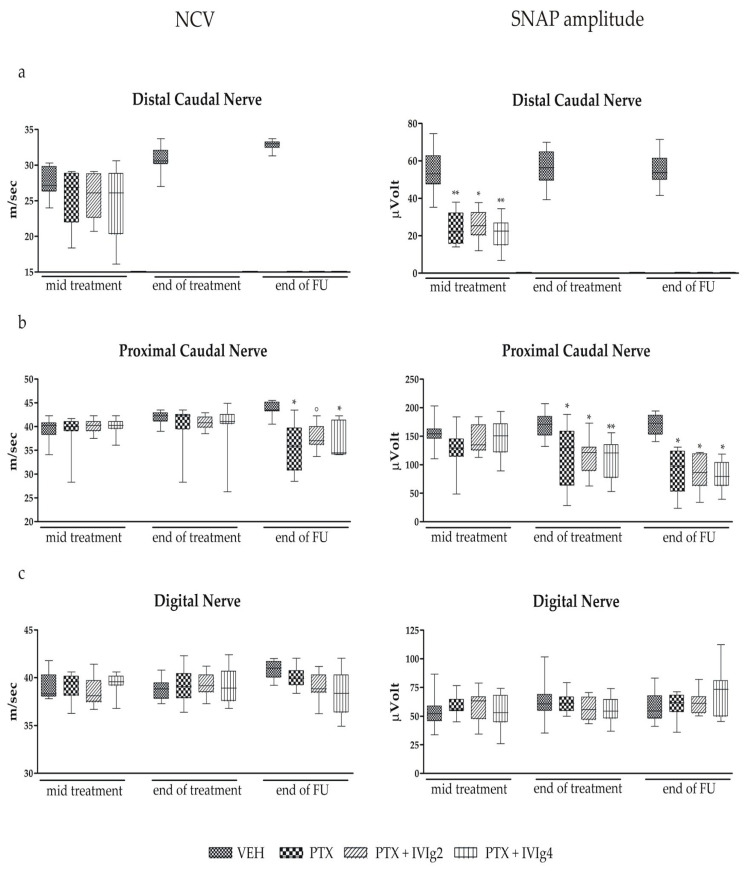
Effects of the IVIg on sensory nerve conduction velocity (NCV) and sensory nerve action potential (SNAP) amplitudes. (**a**) Proximal caudal nerve NCV and SNAP amplitude. (**b**) Distal caudal nerve NCV and SNAP amplitude. (**c**) Digital nerve NCV and SNAP amplitude. ◦ *p* < 0.05, * *p* < 0.01, ** *p* < 0.001 vs. VEH (*n* = 12 rats/group). All data were analyzed with the Kruskal-Wallis test.

The neurophysiological studies conducted mid-treatment revealed a reduction in SNAP amplitude recorded in distal caudal nerve, indicating an early PINP onset, in all PTX-treated animals compared to VEH (*p* < 0.001, Figure 3a). Moreover, at the end of treatment and the follow up period, it was not possible to record any traces from the distal portions of caudal nerves in treated animals, which is compatible with a severe axonal damage and abundant loss of fibers (Figure 3a); these data were, in fact, confirmed by neuropathological examination. At the end of treatment, all PTX-treated groups revealed a significant decrease in proximal caudal SNAP amplitude (*p* < 0.01 and *p* < 0.001), whereas no significant effects were observed in NCV. At the end of follow up, a significant decrease in proximal caudal SNAP amplitude and NCV was observed in PTX-treated rats. The analysis performed in PTX and IVIg co-treated animals did not show any significant protective effects of IVIg administration on neurophysiological parameters (Figure 3b). At any time points, no statistically significant differences were observed in digital nerve action potential amplitude and NCV in all groups of treatment (Figure 3c).

### 2.4. IVIg Reduced the Loss of IENF Induced by PTX

**Figure 4:**
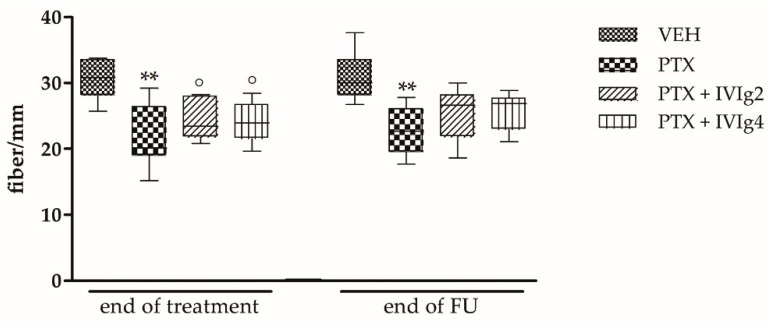
Effects of PTX and IVIg co-treatment on IENF density. IVIg reduces the loss of unmyelinated fibers observed in paclitaxel-treated rats at the end of follow up period. ◦ *p* < 0.05, ** *p* < 0.001 vs. VEH (*n* = 12 rats/group). Data were analyzed using the non-parametric Kruskal-Wallis test.

At the end of the treatment, the animals treated with PTX alone or in combination with IVIg showed a significant reduction in IENF density compared to VEH-treated rats (*p* < 0.001 and *p* < 0.05, respectively; Figure 4).

After the follow up period, only the group treated with PTX alone still showed a decrease of IENF density. In fact, in the PTX + IVIg2 and PTX + IVIg4 groups, a recovery of small unmyelinated fibers density was observed, and the measured density was not significantly different compared to the one observed in specimens collected from VEH-treated rats (Figure 4).

### 2.5. IVIg Reduced Axonal Degeneration Induced by PTX in Caudal Nerves

**Figure 5:**
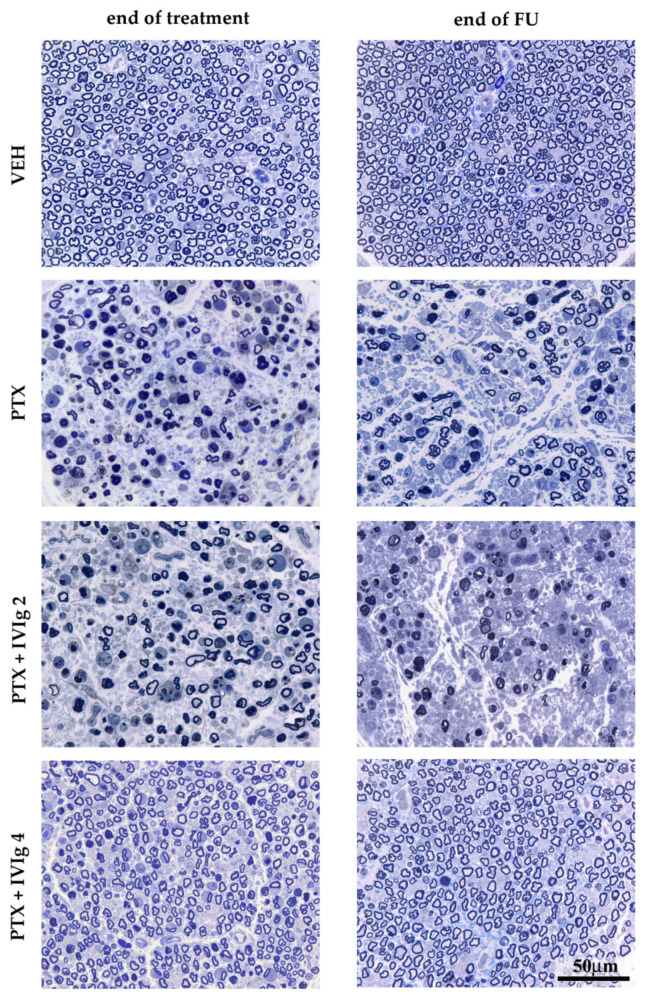
Morphological analysis of distal caudal nerves. IVIg co-treatment starting from the first PTX administration (PTX + IVIg4 group) reduced PTX-induced distal axonal degeneration.

In this part of the study, we analyzed whether IVIg co-treatment was able to revert the distal-to-proximal degeneration pattern typical of PIPN [37]. This analysis was conducted through morphological investigation performed on both distal and proximal caudal nerve segments. In distal caudal nerves, a large number of degenerated fibers were observed in PTX-treated rats both at the end of treatment and follow up period. Similar alterations were observed in proximal caudal nerves, although nerve damage was less prominent in proximal segments compared to distal ones, in agreement with the neurophysiological results. At the end of treatment and after the follow-up period, only the PTX + IVIg4 group showed a reduction in the severity of nerve fiber degeneration in distal caudal nerves vs. PTX alone (Figure 5). However, in proximal caudal nerves, the overall extent of the damage was milder and no effects of the administration of IVIg could be detected. At both experimental time points under investigation, no morphological differences were observed in sciatic nerve and DRG samples of PTX and PTX + IVIg co-treated rats compared to VEH.

### 2.6. IVIg Induced a Reduction of PTX-Induced Macrophages Infiltration

**Figure 6:**
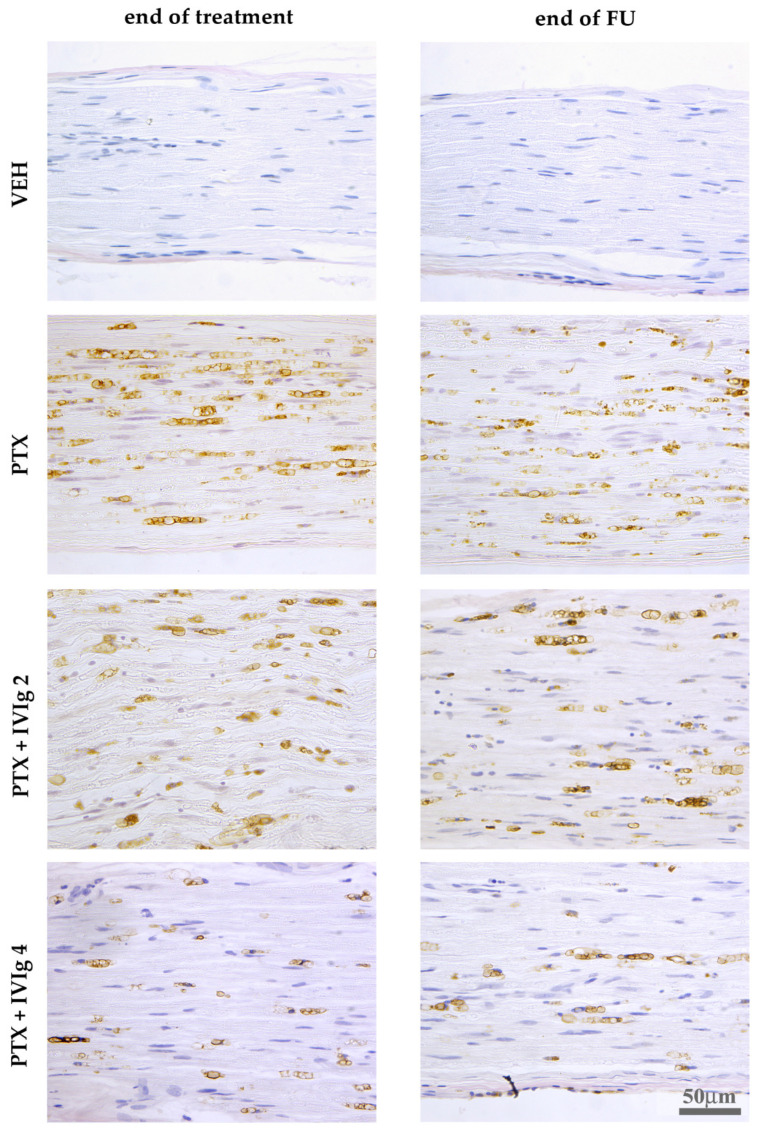
Immunolocalization of CD68^+^ macrophages in distal caudal nerves. IVIg + PTX co-treatment starting from the first day of PTX chemotherapy regimen induced a partial reduction in CD68^+^ infiltrating macrophages at both the end of treatment and after the follow-up period.

At the end of PTX treatment and after the follow-up period, IHC analysis for CD68 evidenced a more robust macrophage infiltration in distal caudal nerves if compared to proximal segments, while no macrophage infiltration was observed in VEH rats. Only the group co-treated with IVIg starting from the first PTX administration (PTX + IVIg4) showed attenuated macrophage infiltration if compared to the PTX group (Figure 6).

No macrophage infiltration was observed in sciatic nerve and DRG samples of PTX and PTX + IVIg co-treated rats compared to VEH.

## 3. Discussion

PIPN is a common and potentially severe adverse event in patients undergoing PTX chemotherapy regimen [2] that may lead to dose reduction or chemotherapy withdrawal. Till now, no neuroprotective agents are recommended for the prevention of CIPN, and only duloxetine is partially effective in decreasing chemotherapy-induced peripheral neuropathic pain in patients with established painful CIPN [38].

In the last decade, activation of immune and immune-like cells in both the peripheral and central nervous systems have gained rising interest as one of the putative mechanisms responsible for PIPN onset and progression. The results of clinical trials indicate that IVIg administration is effective in reducing pain in chronic conditions where neuropathic pain is prominent [39]. Furthermore, their efficacy has already been demonstrated in animal models of autoimmune and inflammatory neuropathies [33,34,35] and in bortezomib-induced peripheral neuropathy [33]. Since no data about their effects in PIPN are available, the results presented in this study fill this gap by reporting that preventive IVIg co-administration could be effective in alleviating PIPN nocifensive behavior and reducing IENF loss after chemotherapy treatment withdrawal. Moreover, IVIg co-treatment is also effective in attenuating PTX-induced axonopathy, which is a very important point since this aspect is a typical feature observed in PIPN-affected patients. Direct evidence that these effects are dependent on the partial reduction of neuroinflammation might be provided by the immunohistochemical analysis performed on distal caudal nerves that show a decrease in macrophage recruitment induced by PTX in animals co-treated with IVIg. This observation is interesting, since it has been demonstrated that PTX administration induces the activation of NLRP3 inflammasome in infiltrated macrophages of DRG and sciatic nerve [24], and this effect might be down-regulated by IVIg.

Furthermore, IVIg could modulate macrophage functions, interfering with the production of the macrophage pro-inflammatory factor involved in peripheral nerve degeneration and exerting their immunomodulatory effects acting on several components of the immune system, as observed in chronic inflammatory demyelinating polyneuropathy [40]. However, these hypotheses are, so far, supported by indirect evidence, and deserve to be further investigated to explain the higher capacity of IVIg to limit macrophages infiltration induced by bortezomib [36] respect to PTX.

Despite a difference at the pathological level, the protective effect of IVIg was not accompanied by an improvement in distal caudal NCV and SNAP, as already observed in the bortezomib-induced peripheral neurotoxicity model [36]. A possible explanation relies on the fact that the protective effect was not sufficiently strong on the largest myelinated fibers providing most of the neurophysiological signals detected with the conventional techniques we used.

In contrast to the protective effects observed, once the animals were co-treated during the entire PTX treatment, the morphological analysis did not show any evident protective effect of IVIg administration, starting at mid treatment of PTX regimen. This lack of effectiveness is not unexpected, since PIPN damage is detectable as early as after two weeks of treatment, as confirmed in experimental PIPN also by the demonstration of a very early increase in neurofilament light chain, a specific biomarker of nerve damage [41] and inflammatory response [42]. However, as reported for the group co-treated with IVIg starting from the first PTX administration, the infusion of IVIg starting at mid treatment of PTX regimen was able to revert mechanical allodynia and IENF loss observed in PTX-treated animals at the end of follow up period. Moreover, it is important to note that, in our PIPN model, we did not observe macrophages infiltration and damage in DRG and sciatic nerves samples of PTX-treated rats at the end of treatment and follow up period. However, we can not exclude that an increase of infiltrating pro-inflammatory macrophages occurs in a time course that matches the onset of the behavioral CIPN phenotype after PTX treatment. In fact, other authors described an increased recruitment and activation of pro-inflammatory macrophages in DRG [15,16,22] and sciatic nerves of PTX-treated animals [16]. These discrepancies may reside in the different animal models used (strain, route of administration, dosage and schedule of treatment). However, our data are in line with other studies reporting that the treatment with microglia/macrophages inhibitor (minocycline) prevents IENF density reduction at the onset of allodynia [43].

## 4. Materials and Methods

### 4.1. Animals and Drugs

Female Wistar rats (175–200 g) were purchased from Envigo Laboratory (Udine, Italy). The animals were housed in an animal facility characterized by a standard light cycle of 12 h on and 12 h off, a constant temperature of 22 °C ± 2, and 50 % ± 20 relative humidity. Rats were housed in an adequate cage (*n* = 2/3 animals per cage) with rodent diet and water ad libitum, subjected to daily monitoring of their clinical conditions and weighted twice a week for a general health check and drug dose adjustment. At each time point of sacrifice, rats were euthanized by CO_2_ inhalation followed by cervical dislocation. All experimental procedures were carried out in accordance with National Institute of Health guidelines for animal care and use of Laboratory animals (DL 2016, Italian Ministry of Health approvation protocol *n* 618/218-PR) and approved by the Milano Bicocca University Ethics Committee (protocol *n* 7579/19).

To generate PIPN, rats received chronic intravenous injection of PTX (PTX, 10 mg/Kg) dissolved in a vehicle solution composed of 10% tween 80, 10% EtOH absolute and 80% saline solution, as previously described in detail [41].

To investigate the therapeutic effect of human immunoglobulins (Ig VENA 50 g/L solution for infusion, ready to use supplied by Kedrion SpA), 48 female Wistar rats were randomized into 4 experimental groups: one group was treated with vehicle for 4 weeks followed by follow up (VEH, *n* = 12), and three groups were treated with PTX alone (PTX, *n* = 12) or in co-treatment with two different IVIg schedules (PTX + IVIg2, *n* = 12; PTX + IVIg4, *n* = 12) followed by follow up. IVIg were infused at the dosage of 1g/kg the day before PTX treatment at the specific time points described in the flow chart of the study (Figure 1a). In detail, IVIg were infused every two weeks, starting from the first PTX infusion (PTX + IVIg4) or PTX mid treatment (PTX + IVIg2) for a total of three and two administrations, respectively. In particular, rats received 4 mL of IVIg in an infusion time of 10 min. The dosage of human IVIg solution selected for the study is equivalent to the daily dose used in patients affected by acute and chronic inflammatory neuropathies, and derives from a previous study in bortezomib-treated animals [36], where their tolerability and pharmacokinetic were demonstrated.

### 4.2. IVIg Serum Levels Determination

For the determination of the levels of IVIg in the serum, blood was collected via the tail vein at baseline, at mid treatment, at the end of treatment and at the end of the follow up period (Figure 1b). Briefly, blood samples were centrifuged at 2200 g for 15 min at room temperature and determined by a commercial nephelometry assay using a IMMAGE 800 device (Beckman Coulter, Brea, CA, USA), as previously described [36].

### 4.3. Nerve Conduction Studies

Neurophysiological assessments were performed at baseline, at mid treatment, two days after the completion of the chemotherapy regimen, and at the end of follow up period (Figure 1a). Sensory nerve conduction velocity (NCV) and sensory nerve action potential (SNAP) for both caudal and digital nerves were obtained using an electromyography apparatus (Myto2 ABN Neuro, Firenze, Italy) according to a previous study [44]. All the recordings were performed orthodromically using stainless steel needle electrodes (Subdermal EEG needle, Ambu™, Ballerup, Denmark). Briefly, through the whole duration of the recording, rats were deeply anaesthetized with volatile isoflurane gas and their body temperature was kept constant at 37 ± 0.5 °C using a heating pad operated via a rectal thermal probe (Harvard Apparatus, Holliston, MA, USA). The caudal nerve was studied in its distal and proximal segments to catch the length dependency typical of PIPN. For the distal stimulation of caudal nerve, the active and reference recording electrodes were placed respectively at 5 and 6 cm from the tail extremity, the stimulating cathode and anode were placed at 2 and 1 cm from the tail extremity, respectively, whereas the ground electrode was placed at 2.5 cm from it. For the proximal stimulation of the caudal nerve, the pair of recording electrodes were placed at 1 and 2 cm from the tail base, the pair of stimulating one at 5 and 6 cm from the tail base, whereas the ground electrode was placed at 2.5 cm from it. For the digital nerve, the active and reference recording electrodes were placed, respectively, near the anklebone and near the patellar bone, the stimulating anode and cathode were placed at the base and at the tip of the fourth toe of the left hind limb, respectively, whereas the ground electrode was placed in the sole.

### 4.4. Assessment of Mechanical Allodynia

Alterations in pain sensitivity in response to mechanical stimuli were assessed using a Dynamic Plantar Aesthesiometer (Ugo Basile Biological Instruments, Varese, Italy), as previously described in detail [44]. These determinations were performed at baseline, at mid treatment, at the end of treatment and at the end of follow up period (Figure 1a).

Briefly, rats were allocated in plexiglass cages placed on a metal grid floor for 15 min. After the acclimation period, a metal filament, exercising a linear increasing force ramp which reaches 50 g in 20 s, was applied to the plantar surface of the hind paw. Mechanical threshold force, i.e., the minimum pressure required to elicit a withdrawal reflex of the paw, was registered three times for each paw and it was automatically recorded by the instrument and then calculated as the average of six consecutive values (expressed in grams). This test was conducted in a controlled behavioral test room by a researcher who was blind to treatments. An upper limit cutoff of 20 s was fixed.

### 4.5. Morphological Analysis

After animal sacrifice sciatic nerves, caudal nerves and DRG samples were collected (Figure 1a) and processed for light microscopy analysis, as previously described [37]. Briefly, semithin sections of 1-μm thickness were prepared from at least two tissue blocks for each animal; they were stained with toluidine blue and examined with a Nikon Eclipse E200 light microscope (Nikon Europe B.V, Amsterdam, The Netherlands).

To evaluate a small-fiber peripheral nerve damage, intra-epidermal nerve fiber (IENF) density in the hind paw footpad was evaluated, as already reported [45]. Samples were collected at the time point indicated in Figure 1a. Briefly, the skin biopsies from the plantar glabrous skin (epidermis and dermis) were obtained from the hind paw footpad of the rats and fixed in PLP 2% (paraformaldehyde-lysine-sodium periodate) for 24 h at 4 °C. The samples were cryoprotected, frozen and serially cut with a cryostat to obtain 20-μm sections. Then, IENF were immunostained with a primary rabbit antibody against PGP 9.5 (GeneTex, Irvine, CA, USA) using a free-floating protocol. To quantify the nociceptive IENF density, the number of nerve fibers that cross the dermal/epidermal junction were counted from three random sections from each sample, and the length of the epidermis was measured. Finally, the density of IENFs was obtained as PGP 9.5 positive cell/length mm.

### 4.6. Immunohistochemical (IHC) Analysis of Infiltrating Cells

To investigate the role of inflammation within DRGs, sciatic and caudal nerves, we determined the CD68 profile, a phagocytically active macrophage detector, on the samples collected after sacrifice as previously described [36]. Briefly, the presence of infiltrating macrophages was evaluated in 3 μm thick sections of Formalin-Fixed Paraffin-Embedded (FFPE) DRGs, sciatic and caudal nerves incubated with anti-CD68 antibody (ED1, mouse anti-rat monoclonal antibody, Abcam, Cambridge, UK).

### 4.7. Statistical Analysis

Statistical analyses of body weight, neurophysiological studies, dynamic test and IENF density were evaluated using the Kruskall-Wallis test followed by Dunn’s post hoc test for each time point. A *p* value < 0.05 was considered statistically significant. All statistical analyses were performed using the GraphPad Prism4 software (GraphPad Software, San Diego, CA, USA).

## 5. Conclusions

The results of our study extend previous observations in the experimental bortezomib-induced peripheral neurotoxicity model [36] and indicates that preventive IVIg co-treatment may not only reduce the nocifensive behavior related to mechanical stimulation, but also the peripheral nerve damage and IENF loss in experimental PIPN. Since axonopathy and behavioral alterations are also common features for other chemotherapeutic agents [46,47], the use of IVIg could potentially be useful, and deserves to be tested, in other similar conditions. We also observed that the neuroinflammatory process seems to be partially involved in the pathogenesis of PIPN, although further in-depth investigations to elucidate the role of IVIg are still needed. However, the identification of specific neuroinflammatory targets in PIPN pathogenesis could help in the development of novel immunomodulating therapies to prevent and/or manage PIPN, improving patient outcomes.

## Data Availability

Data could be found on the Bicocca Open Archive Research Data (BOARD) website of the University of Milano-Bicocca (https://board.unimib.it/research-data/).

## Abbreviations

CIPN	chemotherapy-induced peripheral neuropathy
DRG	dorsal root ganglia
FFPE	formalin-fixed paraffin-embedded
IENF	intra-epidermal nerve fibers
IHC	immunohistochemical
IVIg	human intravenous immunoglobulin
NCV	nerves conduction velocity
NLRP3	nod-like receptor 3 inflammasome
PIPN	paclitaxel-induced peripheral neurotoxicity
PTX	paclitaxel
SNAP	sensory nerve action potential
TLR4	toll-like receptor 4
VEH	vehicle
